# Effects of sugarcane variety and nitrogen application level on the quality and aerobic stability of sugarcane tops silage

**DOI:** 10.3389/fpls.2023.1148884

**Published:** 2023-05-30

**Authors:** Qichao Gu, Lu Zhang, Xiaokang Zhou, Bo Lin, Caixia Zou

**Affiliations:** College of Animal Science and Technology, Guangxi University, Nanning, Guangxi, China

**Keywords:** nitrogen fertilizer, sugarcane tops, N-fixing abilities, fermentation quality, bacterial community, fungal community

## Abstract

To better understand the effects of sugarcane variety and nitrogen application level on silage, we analyzed the fermentation quality, microbial community dynamics, and aerobic exposure of sugarcane tops silage from three sugarcane varieties (B9, C22, and T11) treated with three levels of nitrogen (0, 150, and 300 kg/ha urea). After 132 days of silage, the sugarcane tops silage produced from variety B9, with strong nitrogen fixation ability, treated with nitrogen had the highest crude protein (CP) contents, pH, and yeast counts (*P* < 0.05), as well as the lowest Clostridium counts (*P* < 0.05), and the CP increased with increasing N application level (*P* < 0.05). In contrast, the sugarcane tops silage produced from variety C22, with poor nitrogen fixation ability, treated with 150 kg/ha nitrogen had the highest lactic acid bacteria (LAB) counts, dry matter (DM), organic matter (OM) and lactic acid (LA) contents (*P* < 0.05), as well as the lowest acid detergent fiber (ADF) and neutral detergent fiber (NDF) contents (*P* < 0.05). However, these results were not present in the sugarcane tops silage produced from variety T11, with no nitrogen fixation ability, whether it was treated with nitrogen or not; although the silage was treated with 300 kg/ha nitrogen, the ammonia-N (AN) content was the lowest (*P* < 0.05). After 14 days of aerobic exposure, *Bacillus* abundance increased in the sugarcane tops silage produced from variety C22 treated with 150 kg/ha nitrogen and from varieties C22 and B9 treated with 300 kg/ha nitrogen, while *Monascus* abundance increased in the sugarcane tops silage produced from varieties B9 and C22 treated with 300 kg/ha nitrogen and from variety B9 treated with 150 kg/ha nitrogen. However, correlation analysis showed that *Monascus* was positively correlated with *Bacillus* irrespective of nitrogen level and sugarcane variety. Our results indicated that sugarcane variety C22, with poor nitrogen fixation ability, treated with 150 kg/ha nitrogen produced the highest sugarcane tops silage quality and inhibited the proliferation of harmful microorganisms during spoilage.

## 1 Introduction

In recent years, the increase in the amount of grass-fed livestock has increased the demand for high-quality forage grass. Silage is a traditional means of preserving the nutritional quality of feed crops and producing animal feed through the anaerobic fermentation of epiphytic lactic acid bacteria (McDonald et al., 1991). Its biggest advantage is its use of a wide range of sources of raw materials (such as sugarcane tops, corn stalks, and other crop straw by-products) (Liu et al., 2013; Ren et al., 2019; Zhang et al., 2019). The production of silage is influenced by epiphytic microorganisms, and the quality of forage silage is affected by another important factor: the chemical composition of the forage itself (such as crude protein content and soluble carbohydrate content) (Pahlow et al., 2003). Research has indicated that through reasonable forage management measures in agricultural production, the nutritional value of forage can be adjusted to promote the production of high-quality silage (Nematpour et al., 2020).

Nitrogen fertilization is one of these important forage management measures, and its main function is to supply the nitrogen that is essential for forage growth (Leghari et al., 2016). Therefore, rational nitrogen fertilization can increase the yield and nutritional value of forage grass, and it can also significantly improve the silage quality (Nematpour et al., 2020; Magar et al., 2021). A previous study showed that after treating maize, millet, and sorghum crops with 112.5 kg/ha N, the DM, ADF, and NDF contents increased in their silage (Nematpour et al., 2021). After treating whole-crop wheat with 150 kg/ha N, the LA content and relative forage value increased in its silage, except for the DM content (Li et al., 2016). Xu et al. (2022) compared the effects of 0, 60, 120, and 180 kg/ha N on the mixed silage of intercropped maize and alfalfa, and it was found that with the increase in nitrogen application levels, the DM content, pH, and ammonia-N content decreased in the mixed silage of maize and alfalfa, while the CP content, water-soluble carbohydrate content, and lactic acid content significantly increased by 16.3%, 17.8%, and 72.9%, respectively. Similarly, Osterholz et al. (2021) reported that in maize interseeded with alfalfa or in solo-seeded maize, increased N application levels (0, 56, 112, 168, 224, 280 kg/ha) increased their silage N increased. Moreover, the ammonia-N content in silage increased after N applications of 60, 120, and 180 kg/ha in cattle grass, but it did not show a linear change (Tremblay et al., 2005). In addition, studies have shown that increased N application may increase the nitrate content in forage grass, which has the potential to inhibit the growth of *Clostridium* in silage environments (Ennik et al., 1980; Pahlow et al., 2003).

Sugarcane (*Saccharum* L.) is a crop that consumes a large amount of N fertilizer. The N in fertilizer has an irreplaceable role in physiological metabolism and dry matter accumulation in sugarcane (Mehnaz, 2011; Robinson et al., 2013). Guangxi, China is characterized by a subtropical monsoon climate, which is extremely suitable for the growth of sugarcane, and it is the main sugarcane producing area in China (Chen et al., 2020). Varieties B9, ROC22 (C22), and T11 are common sugarcane varieties used for sugar production in Guangxi, China. However, Yang et al. (2008) compared the N fixation ability of the introduced Brazilian sugarcane varieties B9, T11, and C22 under the ecological conditions in Guangxi, China, and it was found that the average N fixation rate of B9 was over 26.91%, while T11 and C22 had no and poor N fixation ability, respectively. Recently, one study explored the effects of nitrogen application level (0, 50, 100, 150, and 200 kg/ha) and Italian ryegrass (*Lolium multiflorum Lam.*) varieties (Devis, Hellen, and Trinova) on the quality of silage and found that the chemical composition of different forage varieties changed with the change in nitrogen application level, eventually leading to different silage quality (Ertekin et al., 2021). However, even though sugarcane tops are one of the main by-products of sugarcane, there is no report on the effect of differences in the chemical composition of sugarcane tops on their silage quality caused by differences in nitrogen fixation of sugarcane under different nitrogen application levels. Additionally, studies have confirmed that analyzing bacterial and fungal communities can reveal the fermentation process following aerobic exposure of silage (Zhang et al., 2019; Zhang et al., 2022).

Therefore, the main aim of this study was to investigate the effects of differences in the chemical compositions of sugarcane tops, particularly the CP content, on the silage quality and aerobic stability of varieties with different N-fixing abilities in Guangxi, China, under different N application levels. Based on this difference, the changes in the microbial community of sugarcane tops silage after aerobic exposure were also discussed.

## 2 Materials and methods

### 2.1 Silage materials and silage production

Sugarcane tops from different sugarcane varieties treated with different levels of nitrogen were harvested on January 16, 2019 from experimental fields at Guangxi University (E108° 17’, N22° 50’). Three levels of nitrogen application and three sugarcane varieties were included in this study. The three nitrogen application levels were L (0 kg/ha urea), M (150 kg/ha urea), and H (300 kg/ha urea). The three sugarcane varieties were B9 (B), C22 (C), and T11 (T). The plant materials were chopped to a theoretical cut length of 2 cm with a forage cutter (model zfd5570, Zheng Feng Machinery Company, China). After mixing, an average of 1.998 kg of material per group was manually packed into a 2.5-L plastic bottle with a plastic cover. The plastic cover was sealed with two layers of polyethylene film, and the bottle was stored for 132 d at an ambient temperature range of 10°C to 35°C.

### 2.2 Aerobic stability

The aerobic stability of the silage was determined according to the method of Nishino and Touno (2005). Silage samples (80 g) from each treatment were removed and put into a 500-mL plastic bottle with compaction at room temperature (25°C ± 2°C), and covered with a layer of gauze. After 2, 6, and 14 d of aerobic exposure, silage samples in each plastic bottle were thoroughly mixed and subsampled for microbial analyses.

### 2.3 Chemical analysis, fermentation profile, and microbial composition of fresh sugarcane tops

Each silage sample (25 g) was thoroughly combined with 225 mL of ringer’s solution, and the mixture was filtered through two layers of cheese cloth. The pH of this extract was directly measured using a pH meter (Mettler Toledo Delta 320; Mettler-Toledo, Greifensee, Switzerland). One aliquot of the filtrate was centrifuged (12 000 × g, 15 min, 4°C) to assess organic acids (lactic acid, butyric acid) and ammonia-N (AN). The lactic acid and ammonia-N contents were measured following the method of Zhang et al. (2019). The other aliquots of filtrates were continuously diluted (dilutions ranged from 10^−1^ to 10^−6^) for microbial counts. Lactic acid bacteria (LAB) were enumerated using De Man, Rogosa, and Sharpe agar (MRS, Beijing Land Bridge Technology, Beijing, China) at 35°C in an anaerobic incubator for 48 h. Yeasts and molds were enumerated using potato dextrose agar (Beijing Land Bridge Technology, Beijing, China) at 25°C for 72 h. Clostridium was cultivated on Tryptose Sulfite Cycloserine Agar Base (Beijing Land Bridge Technology, Beijing, China) at 37°C for 24 h.

The dry matter (DM) content of fresh and silage samples was determined through air oven drying at 65°C for 72 h. Total nitrogen (TN) was calculated using the Kjeldahl method with crude protein (CP) = TN × 6.25. The non-protein nitrogen (NPN) content was measured by the tungstic acid method described by Licitra et al. (1996). The ash content was measured through high-temperature furnace burning, and the acid detergent fiber (ADF) and neutral detergent fiber (NDF) contents were measured following the method of Zhang et al. (2019) with an ANKOM 200I fiber analyzer (ANKOM Technology, Macedon, NY, USA). The WSC content was determined *via* anthrone sulfuric acid colorimetry (Ren et al., 2019).

### 2.4 DNA extraction, PCR amplification, and sequencing

For sequencing, the sugarcane tops silage and aerobic exposure-silage treated with three standard levels of nitrogen application [L (0 kg/ha urea), M (150 kg/ha urea), H (300 kg/ha urea)] and two varieties (B9 and C22) were selected for microbial analyses. Thus, the groups were as follows: B9×L (BL), B9×M (BM), and B9×H (BH); C22×L (CL), C22×M (CM), and C22×H (CH). These varieties were chosen because the nitrogen fixation ability was different from the previous research background. In addition, analysis of the chemical composition of fresh sugarcane tops and sugarcane tops silage showed that the difference in crude protein contents and silage quality between B9 and C22 was the most pronounced. DNA extraction was conducted according to the methods of Zhang et al. (2019).

Microbial DNA extracted from the silage samples was sent to Huada Genome Sequencing Company (Shenzhen, China) for 16S RNA and ITS-1 gene amplicon sequencing on an Illumina MiSeq PE250 platform. Amplification sequencing of the bacterial 16S RNA was performed using primers 338F (5′-ACTCCTACGGGAGGCAGCAG-3′) and 806R (5′-GGACTACHVGGGTWTCTAAT-3′). Amplification sequencing of fungal ITS was performed using primers ITS1F (5′-CTTGGTCATTTAGAGGAAGTAA-3′) and ITS2-2043R (5′-GCTGCGTTCTTCATCGATGC-3′). The amplification of the bacterial 16S RNA and fungal ITS-1 was conducted according to the methods of Ren et al. (2019) and Romero et al. (2017), respectively. The analyses of 16S rRNA sequencing data and fungal ITS sequencing data were performed as described by Ren et al. (2019) and Romero et al. (2017), respectively. The results were analyzed using the software platform QIME 2.0. Microbial relative abundance maps and correlation maps were drawn using the “ggpolt2” package in RStudio (4.0.3). Bacterial and fungal raw sequencing data have been deposited in the NCBI (https://www.ncbi.nlm.nih.gov/) BioProject database, with accession numbers PRJNA895585 and PRJNA895721, respectively.

### 2.5 Statistical analysis

Statistical analyses were performed in SPSS 19.0 software (SPSS, Chicago, Illinois, USA) for Windows. A one-way analysis of variance (ANOVA) was performed on each index of the fresh sugarcane tops and silage, and a general linear model (GLM) was used for two-way analysis of variance on N levels, sugarcane variety, and their interaction. After aerobic exposure, a GLM was used to perform three-way analysis of variance with N levels, sugarcane variety, and aerobic exposure time as fixed factors and replication as a random factor to analyze bacterial and fungal diversity (OTUs, Shannon, Simpson, Chao, and ACE indexes) of the sugarcane tops silage. Tukey’s HSD test was used to identify significant differences between treatment means. At *P* < 0.05, the effect was deemed significant.

## 3 Results

### 3.1 Characteristics of fresh sugarcane tops

The chemical compositions of the three varieties of fresh sugarcane tops are presented in **Table 1**. With increasing N levels, the CP content of the fresh B9 sugarcane tops was significantly increased (*P* < 0.01). At all N application levels, the CP content in the B9 sugarcane tops was significantly greater than that of C22 and T11 (*P* < 0.01). The WSC contents of the fresh sugarcane tops were significantly affected by the sugarcane variety and the interaction between sugarcane varieties and N levels (*P* < 0.01). Additionally, the WSC contents of the fresh T11 sugarcane tops treated with 0 kg/ha N were significantly higher than those of the B9 and C22 sugarcane tops (*P* < 0.01). After treatment with 150 kg/ha N, the WSC content of fresh C22 sugarcane tops was significantly higher than that of B9 and T11 (*P* < 0.01). However, the WSC content did not differ significantly among sugarcane tops treated with 300 kg/ha N. Similarly, the WSC content did not differ significantly among groups, irrespective of N level. Additionally, the DM, NDF and ADF contents of the fresh sugarcane tops did not differ significantly among varieties or N levels. However, varieties or N levels showed a clear effect on the ash and OM contents of the fresh sugarcane tops (*P* < 0.01). Moreover, the ash content was significantly decreased (*P* < 0.01), while the OM content was significantly increased (*P* < 0.01) in C22 sugarcane tops as the N levels increased.

**Table 1:** Chemical composition of the fresh sugarcane tops.

Treatment	V	Items						
		DM (g/kg FM)	CP (g/kg DM)	WSC (g/kg DM)	NDF (g/kg DM)	ADF (g/kg DM)	Ash (g/kg DM)	OM (g/kg DM)
L	B	253.16^ab^	55.30^d^	50.59^c^	615.90^b^	323.22^b^	73.40^e^	926.60^c^
	C	251.70^ab^	53.25^e^	45.00^c^	655.70^ab^	353.70^ab^	78.70^b^	921.30^f^
	T	218.20^b^	48.96^f^	61.74^b^	627.20^ab^	329.24^ab^	85.50^a^	914.50^g^
M	B	263.10^a^	56.52^c^	66.04^b^	632.90^ab^	352.77^ab^	68.60^g^	931.40^a^
	C	258.50^ab^	55.41^d^	78.80^a^	644.20^ab^	344.71^ab^	75.10^d^	924.90^d^
	T	238.00^ab^	52.54^e^	63.02^b^	632.20^a^	331.13^ab^	76.90^c^	923.10^e^
H	B	262.90^a^	65.52^a^	81.13^a^	642.40^ab^	339.65^ab^	73.60^e^	926.40^c^
	C	281.10^a^	62.20^b^	79.12^a^	544.60^ab^	309.50^ab^	70.50^f^	929.50^b^
	T	270.60^a^	55.37^d^	81.86^a^	687.70^a^	380.50^a^	78.90^b^	921.10^f^
SEM		12.72	0.12	2.91	2.63	1.74	0.29	0.29
P-value								
N		0.11	<0.01	0.21	0.93	0.94	<0.01	<0.01
V		0.05	<0.01	<0.01	0.39	0.58	<0.01	<0.01
N*V		0.20	<0.01	0.01	0.07	0.05	<0.01	<0.01

L, N application of 0 kg/ha; M, N application of 150 kg/ha; H, N application of 300 kg/ha.V, sugarcane variety; B, sugarcane variety B9; C, sugarcane variety C22; T, sugarcane variety T11. DM, dry matter; FM, fresh matter; CP, crude protein; WSC, water-soluble carbohydrates; NDF, neutral detergent fiber; ADF, acid detergent fiber; OM, organic matter. SEM, standard error of the mean. N, N level; V, sugarcane variety; N*V, the interaction between nitrogen level and sugarcane variety. ^a–g^Means within a column without a common superscript letter differ. P < 0.05.

### 3.2 Chemical composition of sugarcane tops silage

The chemical compositions of the sugarcane tops silage are shown in **Table 2**. The DM content in the CM group was significantly higher than that in the TM group (*P*<0.05) and was significantly higher in the CH group compared to the BH and TH groups (*P* < 0.05). Within the same variety, higher N application levels yielded higher contents of CP. Moreover, the CP content in the BL group was significantly higher than that in the TL group (*P* < 0.01) and was significantly higher in the BH group compared to the CH and TH groups (*P* < 0.05). In addition, the WSC content in the TL group was significantly higher than that in the BL and CL groups (*P* < 0.01) and was significantly higher in the CM group compared to the BM and TM groups (*P*<0.01). By contrast, the WSC content did not differ significantly among groups, irrespective of N level. At all N application levels, the NDF and ADF contents of the sugarcane tops silage produced from C22 were lower than those of B9 and T11. Additionally, varieties or N levels showed a clear effect on the ash and OM contents of the sugarcane tops silage (*P* < 0.01). At N application rates of both 0 and 150 kg/ha, the ash content of the sugarcane tops silage produced from T11 was significantly higher than that of B9 and C22 (*P* < 0.01), whereas the OM content of the sugarcane tops silage produced from T11 was significantly lower than those groups (*P* < 0.01). Conversely, at a N application rate of 300 kg/ha, the ash content of the sugarcane tops silage produced from C22 was significantly lower than that of B9 and T11 (*P* < 0.01), whereas the OM content of the sugarcane tops silage produced from C22 was significantly higher than those groups (*P* < 0.01). The NPN contents of the sugarcane tops silage differed according to variety (*P* < 0.05). Moreover, only at a N application rate of 0 kg/ha, the NPN content of the sugarcane tops silage produced from C22 was significantly higher than that of T11 (*P* < 0.05).

**Table 2:** Nutritional composition of the sugarcane tops silage (g/kg DM).

Treatment	V	Items							
		DM	CP	WSC	NDF	ADF	OM	Ash	NPN
L	B	223.18^bc^	57.25^ab^	25.30^c^	616.07^a^	346.32^a^	921.52^b^	78.48^b^	21.02^ab^
	C	214.56^c^	54.52^bc^	22.50^c^	482.39^cd^	270.33^de^	920.45^b^	79.55^b^	22.51^a^
	T	217.04^c^	53.37^c^	30.87^b^	614.39^a^	338.96^a^	913.04^c^	86.96^a^	15.78^b^
M	B	235.82^ab^	58.22^ab^	33.02^b^	613.37^a^	336.37^ab^	929.50^a^	70.50^c^	21.34^a^
	C	247.42^a^	56.25^abc^	39.40^a^	441.31^d^	239.61^e^	931.19^a^	68.81^c^	21.14^ab^
	T	224.20^bc^	54.61^bc^	31.51^b^	592.89^ab^	330.66^abc^	922.86^b^	77.14^b^	17.24^ab^
H	B	217.74^c^	59.19^a^	40.57^a^	550.60^abc^	289.60^cd^	921.37^b^	78.63^b^	19.83^ab^
	C	242.38^a^	58.81^a^	39.56^a^	446.64^d^	247.01^e^	931.01^a^	69.00^c^	19.77^ab^
	T	218.84^bc^	56.15^abc^	40.93^a^	528.37^bc^	294.43^bcd^	917.03^bc^	82.97^ab^	19.64^ab^
SEM		5.50	0.12	2.84	2.23	1.36	0.20	0.20	1.63
P-value									
N		<0.01	0.02	0.46	0.01	0.01	<0.01	<0.01	0.99
V		0.01	0.01	<0.01	<0.01	<0.01	<0.01	<0.01	0.03
N*V		0.05	0.89	0.01	0.50	0.37	0.14	0.14	0.35

L, N application of 0 kg/ha; M, N application of 150 kg/ha; H, N application of 300 kg/ha. V, sugarcane variety; B, sugarcane variety B9; C, sugarcane variety C22; T, sugarcane variety T11. DM, dry matter; CP, crude protein; WSC, water-soluble carbohydrates; NDF, neutral detergent fiber; ADF, acid detergent fiber; OM, organic matter; NPN: non-protein nitrogen. SEM, standard error of the mean. N, N level; V, sugarcane variety; N*V, the interaction between nitrogen level and sugarcane variety. ^a–g^Means within a column without a common superscript letter differ. P < 0.05.

### 3.3 Fermentation indexes and microbial abundance of sugarcane tops silage

The fermentation index and microbial abundance of the sugarcane tops silage are shown in **Table 3**. The lactic acid contents of all untreated groups (N application rate of 0 kg/ha) were significantly lower than that of nitrogen treatment groups. Additionally, at nitrogen application rates of 150 or 300 kg/ha, the pH of the sugarcane tops silage produced from B9 was significantly higher than that of C22 (*P* < 0.05), while the LA content of the sugarcane tops silage produced from C22 was significantly higher than that of B9 and T11 (*P* < 0.05). Furthermore, at a N application rate of 150 kg/ha, the LAB and mold counts of the sugarcane tops silage produced from C22 were higher than those of B9 and T11, but the mold counts of the silage from C22 were below 4.5 log10 cfu/g FM. However, at N application rates of both 0 and 150 kg/ha, the Clostridium counts of the sugarcane tops silage produced from B9 were significantly lower than that of C22 and T11 (*P* < 0.01). In addition, at a N application rate of 300 kg/ha, the AN content of the sugarcane tops silage produced from T11 was significantly lower than that of B9 and C22 (*P* < 0.05), while the yeast counts of sugarcane tops silage produced from B9 were higher than those of C22 and T11 (*P* < 0.01).

**Table 3:** Fermentation indexes and microbial counts in the sugarcane tops silage.

Treatment	V	Fermentation index (g/kg DM)			Microbial counts (log10 cfu/g FM)				
		pH	LA	AN	LAB	Clostridium	Yeast	Mold	
L	B	4.47^a^	11.45^c^	0.59^a^	3.50^e^	3.57^d^	4.17^ab^	3.50^cd^	
	C	4.45^a^	17.37^bc^	0.44^ab^	4.16^bc^	3.99^bc^	4.09^b^	3.29^e^	
	T	4.39^ab^	29.40^bc^	0.50^ab^	4.03^c^	3.87^c^	4.15^ab^	3.79^b^	
M	B	4.18^abc^	58.80^b^	0.38^bc^	4.45^b^	3.93^bc^	4.00^bc^	3.33^e^	
	C	3.81^d^	111.95^a^	0.38^bc^	4.98^a^	5.03^a^	3.80^de^	4.21^a^	
	T	4.04^cd^	55.02^b^	0.36^bc^	3.74^d^	4.97^a^	3.69^e^	3.21^e^	
H	B	4.12^c^	54.20^b^	0.49^ab^	4.44^b^	3.92^bc^	4.31^a^	3.63^bc^	
	C	3.82^d^	99.05^a^	0.45^ab^	4.35^b^	4.01^bc^	3.90^cd^	3.63^bc^	
	T	3.90^cd^	47.19^bc^	0.19^c^	4.62^ab^	4.17^b^	3.91^cd^	3.64^bc^	
SEM		0.06	7.19	0.03	0.37	0.50	0.20	0.30	
P-value									
N		<0.01	<0.01	0.02	0.88	<0.01	<0.01	0.11	
V		0.02	0.01	0.02	<0.01	<0.01	<0.01	<0.01	
N*V		0.33	0.09	0.04	<0.01	<0.01	0.04	<0.01	

L, N application of 0 kg/ha; M, N application of 150 kg/ha; H, N application of 300 kg/ha. V, sugarcane variety; B, sugarcane variety B9; C, sugarcane variety C22; T, sugarcane variety T11. LA, lactic acid; AN, NH_3_-N; LAB, Lactic acid bacteria. SEM, standard error of the mean. N, N level; V, sugarcane variety; N*V, the interaction between nitrogen level and sugarcane variety. ^a–e^Means within a column without a common superscript letter differ. P < 0.05.

### 3.4 Bacterial and fungal diversity of sugarcane tops silage after aerobic exposure

The bacterial alpha diversity of the sugarcane tops silage after aerobic exposure is shown in **Table 4**. The Good’s coverage for all groups was approximately 1, indicating that the bacterial community analysis conducted at this sequencing depth was reliable. Nitrogen levels and aerobic exposure time had a significant effect on the operational taxonomic units (OTUs) and Shannon, Simpson, Chao, and ACE indexes (*P* < 0.01), while varieties only had a significantly effect on the OTUs and Shannon Simpson and indexes (*P* < 0.01). For all groups, the OTUs and the Chao and ACE indexes decreased on days 0–6 of aerobic exposure, but increased on days 6–14. Additionally, after 2 days of aerobic exposure, the OTUs and Shannon, Simpson, Chao, and ACE indexes were greatest for the BL group. By contrast, after 6 days of aerobic exposure, the OTUs and the Chao and ACE indexes were greatest for the CM group, while the Shannon and Simpson indexes were greatest for the CL group. However, after 14 days of aerobic exposure, the OTUs and the Chao and ACE indexes were once again greatest for the BL group, and the Shannon index was greatest for the CH group.

**Table 4:** Bacterial alpha diversity in sugarcane tops silage produced from different varieties and treated with different levels of nitrogen after aerobic exposure.

AE (days)	Treatment	V	OTUs	Shannon	Simpson	Chao	ACE	Coverage
0	B	L	260.67^ab^	3.92^abcd^	0.89^a^	236.50^a^	243.17^ab^	1
		M	243.00^abcd^	3.82^abcde^	0.89^a^	178.13^abcdef^	196.79^abcdefg^	1
		H	266.00^ab^	3.72^abcdef^	0.87^a^	187.50^abcde^	201.57^abcdef^	1
	C	L	255.33^abc^	4.23^ab^	0.91^a^	219.61^abc^	231.33^abc^	1
		M	226.33^abcde^	2.75^ghijk^	0.69^bc^	212.86^abcd^	226.93^abcde^	1
		H	179.33^cdef^	1.52^l^	0.35^e^	228.86^abc^	193.92^abcdefg^	1
2	B	L	264.00^ab^	4.06^abc^	0.90^a^	211.96^abcd^	227.77^abcd^	1
		M	202.00^bcdef^	3.69^abcdef^	0.89^a^	160.08^cdef^	175.98^cdefgh^	1
		H	197.67^bcdef^	3.17^defghi^	0.82^ab^	157.57^def^	161.93^defgh^	1
	C	L	188.00^bcdef^	3.94^abcd^	0.89^a^	144.50^def^	148.70^fgh^	1
		M	163.33^ef^	3.03^fghij^	0.80^ab^	123.05^ef^	130.53^gh^	1
		H	199.67^bcdef^	2.04^kl^	0.54^d^	162.55^bcdef^	172.18^cdefgh^	1
6	B	L	165.67^def^	3.28^cdefg^	0.82^ab^	146.70^def^	144.94^fgh^	1
		M	142.67^f^	3.06^efghij^	0.80^ab^	117.18^f^	122.02^h^	1
		H	143.33^f^	2.46^ijk^	0.69^bc^	111.95^f^	126.11^h^	1
	C	L	180.33^cdef^	3.51^bcdefg^	0.86^a^	138.79^ef^	153.08^fgh^	1
		M	187.00^bcdef^	2.11^kl^	0.60^cd^	158.98^cdef^	159.60^fgh^	1
		H	148.00^e^	2.42^jk^	0.68^bc^	120.53^ef^	118.58^h^	1
14	B	L	289.67^a^	3.63^abcdef^	0.84^a^	235.60^a^	246.73^a^	1
		M	224.33^abcde^	3.50^bcdefg^	0.84^a^	169.34^abcdef^	180.23^bcdefgh^	1
		H	196.67^bcdef^	4.32^a^	0.91^a^	152.95^cdef^	160.32^egh^	1
	C	L	256.33^abc^	3.97^abc^	0.88^a^	230.54^a^	222.02^abcdef^	1
		M	224.00^abcde^	3.74^abcdef^	0.83^a^	200.37^abcd^	204.20^abcdef^	1
		H	187.00^bcde^	4.41^a^	0.90^a^	158.79^cdef^	162.00^defgh^	1
SEM			6.29	0.10	0.02	5.76	5.66	0
P-value								
N			<0.01	<0.01	<0.01	<0.01	<0.01	NS
V			0.08	<0.01	<0.01	0.73	0.51	NS
T			<0.01	<0.01	<0.01	<0.01	<0.01	NS
N*T			0.51	<0.01	<0.01	0.24	0.47	NS
V*T			0.11	<0.01	<0.01	0.11	0.14	NS
N*V			0.58	<0.01	<0.01	0.08	0.15	NS
N*V*T			0.37	<0.01	<0.01	0.84	0.49	NS

AE, aerobic exposure time. L, N application of 0 kg/ha; M, N application of 150 kg/ha; H, N application of 300 kg/ha. V, sugarcane variety; B, sugarcane variety B9; C, sugarcane variety C22; T, sugarcane variety T11. OTUs, operational taxonomic units. SEM, standard error of the mean. N, N level; V, sugarcane variety; T, aerobic exposure time; N*T, interaction between nitrogen levels and aerobic exposure time; V*T, interaction between sugarcane variety and aerobic exposure time; N*V, the interaction between nitrogen levels and sugarcane variety. N*V*T, the interaction among nitrogen levels, sugarcane variety, and aerobic exposure time. NS, not significantly. ^a–l^Means within a column without a common superscript letter differ. P < 0.05.

The fungal alpha diversity of the sugarcane tops silage after aerobic exposure is shown in **Table 5**. The Good’s coverage of each group was approximately 1, indicating that the fungal community analysis conducted at this sequencing depth was reliable. Nitrogen levels and aerobic exposure time had a significant effect on the OTUs and the Shannon, Simpson, Chao, and ACE indexes (*P*<0.01), while varieties had no significant effect on the Shannon and Simpson indexes. Across all groups, the OTUs and the Shannon, Chao, and ACE indexes increased on days 0–2 of aerobic exposure and decreased on days 2–14. In addition, the OTUs and the Chao and ACE indexes were greatest for the CH group on day 0 of aerobic exposure, followed by the BL group. However, on days 2, 6, and 14 of aerobic exposure, the OTUs and the Chao index were highest for the CL group, especially on day 14 of aerobic exposure, and the Shannon and Simpson indexes were also highest for this group. Moreover, the ACE index was highest for the CL group on days 2 and 14 of aerobic exposure, but on day 6 of aerobic exposure, the ACE index was highest for the BM group.

**Table 5:** Fungal alpha diversity in sugarcane tops silage produced from different varieties and treated with different levels of nitrogen after aerobic exposure.

AE (days)	Treatment	V	OTUs	Shannon	Simpson	Chao	ACE	Coverage
0	B	L	551.33^abcdef^	4.71^ab^	0.78^abcd^	542.59^abcd^	539.20^abcde^	1
		M	526.00^abcdef^	5.33^ab^	0.85^abc^	495.64^abcd^	515.97^abcde^	1
		H	550.00^abcdef^	5.90^a^	0.95^ab^	540.01^abcd^	537.19^abcde^	1
	C	L	411.67^cdefg^	5.53^a^	0.91^ab^	430.75^bcde^	394.40^cdef^	1
		M	381.67^defg^	4.81^ab^	0.83^abc^	373.81^cde^	362.48^cdef^	1
		H	860.67^a^	5.88^a^	0.95^ab^	847.68^a^	868.00^a^	1
2	B	L	798.67^abc^	6.04^a^	0.95^ab^	810.17^ab^	816.74^ab^	1
		M	639.00^abcd^	6.30^a^	0.97^a^	603.71^abc^	618.62^abcd^	1
		H	684.67^abcd^	4.99^ab^	0.85^abc^	644.63^abc^	666.10^abc^	1
	C	L	901.33^a^	6.20^a^	0.96^ab^	852.90^a^	896.77^a^	1
		M	606.00^abcde^	5.57^a^	0.91^ab^	612.58^abc^	617.97^abcde^	1
		H	210.67^efg^	2.24^cd^	0.53^cdef^	191.59^de^	205.92^df^	1
6	B	L	689.33^abcd^	4.99^ab^	0.91^ab^	680.68^abc^	695.44^abc^	1
		M	792.67^abc^	5.10^ab^	0.91^ab^	827.07^ab^	844.50^ab^	1
		H	351.67^defg^	2.17^cd^	0.60^abcdef^	372.18^cde^	381.55^cdef^	1
	C	L	832.67^ab^	4.81^ab^	0.88^abc^	828.38^ab^	843.79^ab^	1
		M	598.33^abcde^	4.15^abc^	0.79^abcd^	662.37^abc^	687.31^abc^	1
		H	63.00^g^	1.29^d^	0.45^def^	55.98^e^	60.92^f^	1
14	B	L	447.67^bcdefg^	3.14^bcd^	0.67^abcde^	427.37^bcde^	428.51^bcdef^	1
		M	404.00^cdefg^	3.20^bcd^	0.59^bcdef^	368.96^cde^	388.73^cdef^	1
		H	168.67^fg^	1.25^d^	0.37^ef^	146.34^de^	179.82^ef^	1
	C	L	664.33^abcd^	5.06^ab^	0.89^ab^	667.70^abc^	670.06^abc^	1
		M	311.00^defg^	1.32^d^	0.30^f^	355.60^cde^	357.19^cdef^	1
		H	65.00^g^	1.19^d^	0.37^ef^	54.90^e^	61.15^f^	1
SEM			34.66	0.23	0.03	34.99	35.70	0
P-value								
N			<0.01	<0.01	<0.01	<0.01	<0.01	NS
V			0.24	0.13	0.22	0.38	0.34	NS
T			<0.01	<0.01	<0.01	<0.01	<0.01	NS
N*T			<0.01	<0.01	<0.01	<0.01	<0.01	NS
V*T			0.60	0.38	0.56	0.47	0.60	NS
N*V			0.14	0.03	0.10	0.19	0.19	NS
N*V*T			0.06	0.42	0.59	0.09	0.06	NS

AE, aerobic exposure time. L, N application of 0 kg/ha; M, N application of 150 kg/ha; H, N application of 300 kg/ha. V, sugarcane variety; B, sugarcane variety B9; C, sugarcane variety C22; T, sugarcane variety T11. OTUs, operational taxonomic units. SEM, standard error of the mean. N, N level; V, sugarcane variety; T, aerobic exposure time; N*T, interaction between nitrogen levels and aerobic exposure time; V*T, interaction between sugarcane variety and aerobic exposure time; N*V, the interaction between nitrogen levels and sugarcane variety. N*V*T, the interaction among nitrogen levels, sugarcane variety, and aerobic exposure time. NS, not significantly. ^a–g^Means within a column without a common superscript letter differ. P < 0.05.

### 3.5 Bacterial community dynamics after aerobic exposure

**Figure 1:**
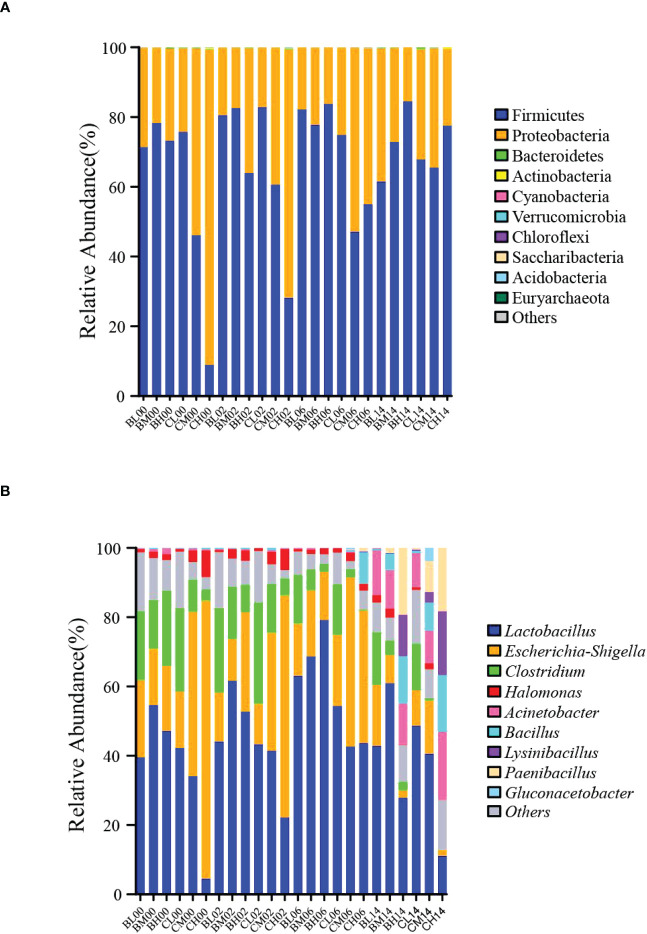
Bacterial communities in sugarcane tops silage during 14 d of aerobic exposure. **(A)** Phylum-level bacterial communities in sugarcane-top silage during 14 d of aerobic exposure; **(B)** Genus-level bacterial communities in sugarcane-top silage during 14 d of aerobic exposure. BL, BM, and BH indicate sugarcane tops silage produced from varieties B9 treated with nitrogen application of 0 kg/ha, 150 kg/ha, and 300 kg/ha, respectively. CL, CM, and CH indicate sugarcane tops silage produced from varieties C22 treated with nitrogen application of 0 kg/ha, 150 kg/ha, and 300 kg/ha, respectively.

The changes in the phylum-level bacterial community after aerobic exposure are shown in **Figure 1A**. Proteobacteria and Firmicutes were the most abundant bacterial phyla on days 0–14 of aerobic exposure in all treatment groups. Furthermore, during early aerobic exposure (days 0–2), the relative abundance of Firmicutes increased in the BM, CM, BL, and CL groups, and that of Proteobacteria decreased. During the middle period of aerobic exposure (days 2–6), the relative abundance of Firmicutes also increased in the BL and BH groups, whereas that of Proteobacteria decreased. However, the relative abundance of Firmicutes in the CM group decreased, and that of Proteobacteria increased during the middle period of aerobic exposure. In contrast, during the later period of aerobic exposure (days 6–14), the relative abundance of Firmicutes in the BM, BL, and CL groups decreased and that of Proteobacteria increased in the three groups. On the contrary, the relative abundance of Firmicutes increased in the CM and BH groups, and that of Proteobacteria decreased during the later period of aerobic exposure. In addition, in the CH group the relative abundance of Firmicutes increased during the 14 days of aerobic exposure, while that of Proteobacteria decreased.

Alterations in bacterial community composition at the genus level after aerobic exposure are shown in **Figure 1B**. On days 0 and 2 of aerobic exposure, the top four dominant bacterial genera in the CH group were *Lactobacillus*, *Escherichia-Shigella*, *Clostridium*, and *Halomonas*. Similarly, these genera were the top four dominant bacterial genera in groups BL, BM, CL, BH, and CM on days 0, 2, and 6 of aerobic exposure. The top four dominant bacterial genera in the CH group were *Lactobacillus*, *Escherichia-Shigella*, *Paenibacillus*, and *Halomonas*, which dominated on day 6 of aerobic exposure. Moreover, after 14 days of aerobic exposure, *Lactobacillus*, *Escherichia-Shigella*, *Clostridium*, and *Acinetobacter* were the top four dominant bacterial general in groups BL, BM, and CL; *Lactobacillus, Lysinibacillius*, *Paenibacillus*, and *Bacillus* were the top four dominant bacterial genera in the CM group; *Lactobacillus*, *Escherichia-Shigella*, *Acinetobacter*, *Paenibacillus* were the top four dominant bacterial genera in the BH group; and *Acinetobacter*, *Lysinibacillius*, *Paenibacillus*, and *Bacillus* were the top four dominant bacterial genera in the CH group. Additionally, the relative abundance of *Lactobacillus* in the BL and BH groups increased on days 0–6 of aerobic exposure but decreased on days 6–14 of aerobic exposure. Similarly, the relative abundance of *Escherichia-Shigella* in the BH group increased on days 0–2 of aerobic exposure but decreased on days 2–14. In addition, during the 14 days of aerobic exposure, the relative abundance of *Acinetobacter* increased in the BL and BM groups. At the same time, the relative abundances of *Lactobacillus* increased in the BM, CL, CM, and CH groups. Furthermore, the relative abundances of *Lysinibacilli*, *Paenibacillus*, and *Bacillus* also increased in the BH and CH groups. In particular, after 14 days of aerobic exposure, the relative abundances of *Lysinibacilli*, *Paenibacillus*, and *Bacillus* were 11.99%, 19.29%, and 13.55%, respectively, in the BH group. Similar to the CM group, the relative abundances of *Bacillus* and *Paenibacillus* increased after 14 days of aerobic exposure. By contrast, in the BM and CL groups, the relative abundances of *Escherichia-Shigella* and *Clostridium* decreased during the 14 days of aerobic exposure. Moreover, on days 0–14 of aerobic exposure, the relative abundance of *Escherichia-Shigella* decreased in the CM and CH groups. The relative abundance of *Clostridium* also decreased in the BH group after 14 days of aerobic exposure.

### 3.6 Fungal community dynamics after aerobic exposure

**Figure 2:**
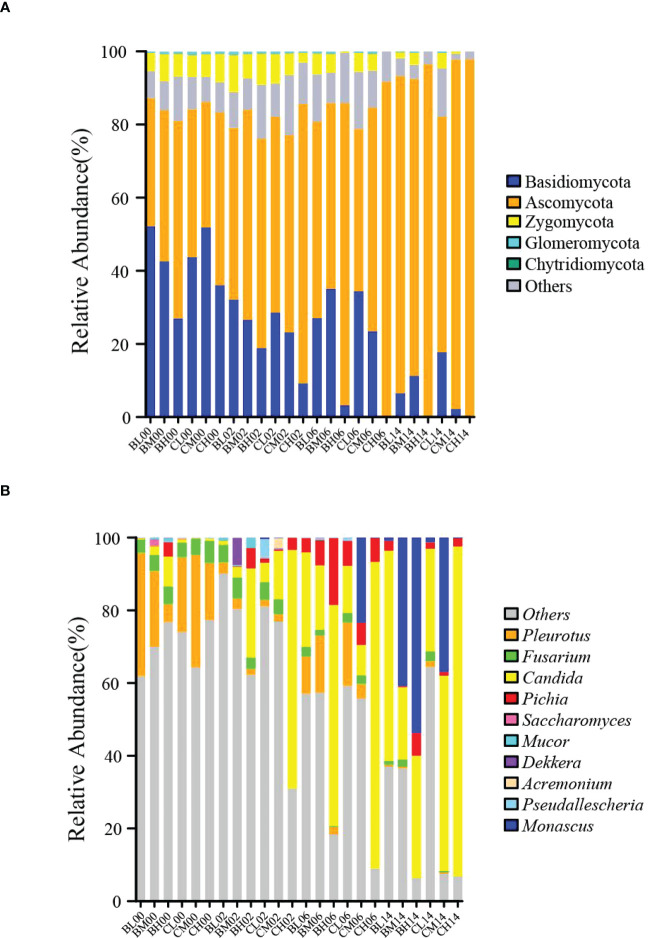
Fungal communities in sugarcane tops silage during 14 d of aerobic exposure. **(A)** Phylum-level fungal communities in sugarcane-top silage during 14 d of aerobic exposure; **(B)** Genus-level fungal communities in sugarcane-top silage during 14 d of aerobic exposure. BL, BM, and BH indicate sugarcane tops silage produced from varieties B9 treated with nitrogen application of 0 kg/ha, 150 kg/ha, and 300 kg/ha, respectively. CL, CM, and CH indicate sugarcane tops silage produced from varieties C22 treated with nitrogen application of 0 kg/ha, 150 kg/ha, and 300 kg/ha, respectively.

The changes in phylum-level fungal community structure after aerobic exposure are shown in **Figure 2A**. Ascomycota and Basidiomycota were the most abundant fungal phyla during the 14 days aerobic exposure. Furthermore, after 14 days of aerobic exposure, the relative abundance of Ascomycota increased and that of Basidiomycota decreased in all six treatment groups.

The changes in genus-level fungal community structure after aerobic exposure are shown in **Figure 2B**. On day 0 of aerobic exposure, *Pleurotus* and *Fusarium* were the top two dominant fungal genera in all groups. By contrast, on day 6 of aerobic exposure, *Candida*, *Pichia*, and *Pleurotus* were the top three dominant fungal genera in groups BL, BM, BH, CL, and CM, while on day 14 of aerobic exposure, *Candida* and *Pichia* were the top two dominant fungal genera in the BL group and *Monascus* and *Candida* were the top two dominant fungal taxa in the BM, BH, and CM groups. Furthermore, on days 2, 6, and 14 of aerobic exposure, *Candida* and *Pichia* were the dominant fungi in the CH group. Additionally, after 14 days of aerobic exposure, the relative abundance of *Monascus* suddenly increased in the BM, BH, and CM groups, and the relative abundance of *Candida* suddenly increased in the BL, BM, CL, and CH groups. In particular, after 14 days of aerobic exposure, the relative abundance of *Monascus* was greatest in the BH group across the six groups with a relative abundance of 53.72%, while the relative abundance of *Candida* was greatest in the CH group with a relative abundance of 90.73%.

### 3.7 Correlations between bacterial and fungal community compositions

**Figure 3:**
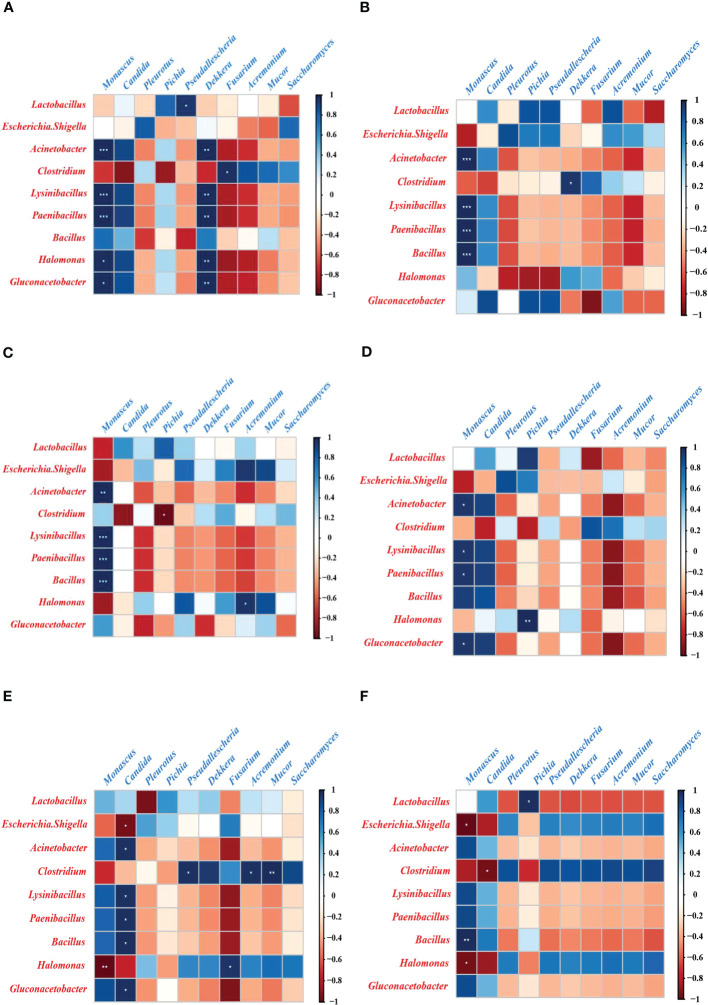
Correlations between bacterial and fungal communities in sugarcane tops silage after aerobic exposure. Fungi are shown in blue text, and bacteria are shown in red text. **(A–F)**. Correlations between bacteria and fungi in the BL **(A)**, BM **(B)**, BH **(C)**, CL **(D)**, CM **(E)**, and CH **(F)** groups after aerobic exposure. BL, BM, and BH indicate sugarcane tops silage produced from varieties B9 treated with nitrogen application of 0 kg/ha, 150 kg/ha, and 300 kg/ha, respectively. CL, CM, and CH indicate sugarcane tops silage produced from varieties C22 treated with nitrogen application of 0 kg/ha, 150 kg/ha and 300 kg/ha, respectively. *: 0.01 < p < 0.05; **p < 0.01; ***p < 0.001.

To clarify the relationship between bacteria and fungi after aerobic exposure in sugarcane tops silage produced from different varieties and treated with different levels of N, Spearman’s correlations were used to analyze the bacterial and fungal communities at the genus level (**Figure 3**, **Supplementary data 1** and **2**). In the BL group, *Monascus* was positively correlated with *Acinetobacter* (r = 1.00, *P* < 0.01), *Lysinibacillus* (r = 1.00, *P* < 0.01), *Bacillus* (r = 0.75, *P* = 0.25), *Halomonas* (r = 1.00, *P =* 0.014), and *Paenibacillus* (r = 1.00, *P* < 0.01) (**Figure 3A**). Moreover, in the BM group, *Monascus* was positively correlated with *Acinetobacter* (r=1.00, *P*<0.01), *Lysinibacillus* (r=1.00, *P*<0.01), *Bacillus* (r = 1.00, *P* < 0.01), *Halomonas* (r = 0.47, *P =* 0.53), and *Paenibacillus* (r = 1.00, *P* < 0.01) (**Figure 3B**). In the BH group, *Monascus* was positively correlated with *Acinetobacter* (r = 0.99, *P* < 0.01), *Lysinibacillus* (r = 1.00, *P* < 0.01), *Bacillus* (r = 1.00, *P* < 0.01), *Halomonas* (r = -0.85, *P =* 0.15), and *Paenibacillus* (r = 1.00, *P* < 0.01) (**Figure 3C**). Similarly, in the CL group, *Monascus* was positively correlated with *Acinetobacter* (r = 0.96, *P =* 0.04), *Lysinibacillus* (r = 0.96, *P =* 0.04), *Bacillus* (r = 0.93, *P* = 0.07), and *Paenibacillus* (r = 0.96, *P =* 0.04), while it was negatively correlated with *Halomonas* (r = −0.34, *P =* 0.66) (**Figure 3D**). In the CM group, *Monascus* was positively correlated with *Acinetobacter* (r = 0.78, *P =* 0.22), *Lysinibacillus* (r = 0.83, *P =* 0.17), *Bacillus* (r = 0.79, *P* = 0.21), *Halomonas* (r = –0.99, *P* < 0.01), and *Paenibacillus* (r = 0.81, *P* = 0.19), while it was negatively correlated with *Halomonas* (r = −0.99, *P* < 0.01) (**Figure 3E**). Also in the CH group, *Monascus* was positively correlated with *Acinetobacter* (r = 0.87, *P* = 0.13), *Lysinibacillus* (r = 0.88, *P =* 0.12), *Bacillus* (r = 1.00, *P* < 0.01), *Halomonas* (r = –0.96, *P =* 0.05), and *Paenibacillus* (r = 0.90, *P =* 0.10), while it was negatively correlated with *Halomonas* (r = −0.96, *P =* 0.05) (**Figure 3F**). Additionally, in the BL group, *Dekkera* was significantly and positively correlated with *Acinetobacter* (r = 1.00, *P* < 0.01) and *Gluconacetobacter* (r = 1.00, *P* < 0.01) (**Figure 3A**). Similarly, in the CL group, *Dekkera* was positively correlated with *Acinetobacter* (r = 0.04, *P =* 0.96) and *Gluconacetobacter* (r = 0.06, *P =* 0.94) (**Figure 3D**). By contrast, in the BM group, *Dekkera* was negatively correlated with *Acinetobacter* (r = –0.37, *P =* 0.63) and *Gluconacetobacter* (r = –0.52, *P =* 0.48) (**Figure 3B**). Furthermore, in the BH group, *Dekkera* was negatively correlated with *Acinetobacter* (r = –0.34, *P =* 0.67) and *Gluconacetobacter* (r = –0.72, *P =* 0.28) (**Figure 3C**). In the CM group, *Dekkera* was negatively correlated with *Acinetobacter* (r = –0.47, *P =* 0.54) and *Gluconacetobacter* (r = –0.50, *P =* 0.50) (**Figure 3E**). In the CH group, *Dekkera* was negatively correlated with *Acinetobacter* (r = –0.29, *P =* 0.71) and *Gluconacetobacter* (r= –0.29, *P=* 0.71) (**Figure 3F**).

## 4 Discussion

### 4.1 Chemical characteristics of fresh sugarcane tops before ensiling

In the current study, the CP content of each experimental group increased with increasing nitrogen levels of up to 300 kg/ha. Similarly, increased N levels led to elevated CP content in Italian ryegrass, whole-crop wheat, dual-purpose barley, and amaranth, and the CP content peaked at a N application rate of 120 kg/ha in Italian ryegrass; at 180 kg/ha in dual-purpose barley; and at 225 kg/ha in whole-crop wheat, while in amaranth, the CP content peaked at a N application rate of 240 kg/ha (Abbasi et al., 2012; Li et al., 2016; Lv et al., 2017; Ertekin, 2022). The result is due to the higher available N content, which in turn supports increased protein synthesis, although the sugarcane present no or poor nitrogen fixation ability (Boddey et al., 2001; Zeng et al., 2020). Moreover, in the present study, the greatest increase in CP content was observed in the fresh B9 sugarcane tops. This is mainly due to the higher nitrogen fixation efficiency of B9 (Yang et al., 2008; Zeng et al., 2020). Additionally, in the present study, sugarcane variety had a very significant effect on the WSC content of the fresh sugarcane tops, among which the WSC contents of the fresh C22 sugarcane tops were the highest after treatment with 150 kg/ha N. Similar changes occurred in different winter wheat cultivars in the early stages and perennial ryegrass cultivars treated with N fertilization, and the WSC content was higher in the winter wheat cultivar Toronto and perennial ryegrass cultivars AberDart and Baronka (Diekmann and Fischbeck, 2005; Conaghan et al., 2012; Olszewska, 2021). One reason for this could be that plant sucrose accumulation is a process of the genetic regulation of complex traits, while different plant varieties have their own genetic traits (Ming et al., 2001). It is worth noting that sufficient WSC is critical for the successful fermentation of lactic acid bacteria. In the current study, the Ash and OM contents were affected by N levels. In C22 sugarcane tops, the Ash content was significantly decreased, while the OM content was significantly increased as N levels increased. Similar results were also found in Italian ryegrass (Ertekin et al., 2021). This may be because plants directly synthesize more N-containing organic compounds in plants by absorbing N from the environment, and plant leaves are its main storage site (Ishikawa et al., 2009; Bohórquez-Sánchez et al., 2023). In contrast, possibly as a result of the differences in plant variety, the OM content decreased as N levels increased in perennial ryegrass and oats (Lovett et al., 2004; Conaghan et al., 2012; Kumari et al., 2014).

### 4.2 Chemical characteristics of sugarcane tops silage

In this study, when the N application rate was 150 or 300 kg/ha, the DM content in the sugarcane tops silage produced from C22 was significantly higher than that of T11. Similarly, after nitrogen application, the different varieties of annual ryegrass and whole-crop maize silage were also affected by the DM content of their silage, and the DM content of the annual ryegrass silage produced from the variety Baquend at N application rates of 225 kg/ha was the highest, while that of the whole-crop maize silage produced by the variety Andante at N application rates of 168 kg/ha was the highest (Lynch et al., 2013; Assouma and Çelen, 2022). Studies have shown that the chlorophyll content of sugarcane leaves is closely related to the accumulation of DM, and at N application rates of 150 or 300 kg/ha, the relative chlorophyll content of C22 sugarcane leaves is significantly higher than that of T11 sugarcane leaves (Zeng et al., 2020). Thus, the main mechanism behind this result is the significant difference in the DM content between plant varieties before ensiling. Meanwhile, this is also confirmed in the present study. In addition, in this study, the contents of NDF and ADF in the sugarcane tops silage produced from variety C22 were significantly lower than the contents of NDF and ADF in sugarcane tops silage produced from varieties B9 and T11 at all levels of N application tested. Similar results were found in annual ryegrass silage and Italian ryegrass silage at any N application level, and the lowest NDF and ADF contents were found in annual ryegrass silage produced from the Elif variety, while the lowest NDF content was found in Italian ryegrass silage produced from the Devis variety (Ertekin et al., 2021; Assouma and Çelen, 2022). A possible reason for this is that the composition and structure of sugarcane cell walls (cellulose, hemicellulose, and lignin) vary among different forage varieties (Figueiredo et al., 2019). Ball et al. (1996) pointed out that when the NDF content in forage grass is greater than 40% and the ADF content is greater than 30%, its feeding value will begin to decline. Furthermore, in the current study, higher N application levels yielded higher contents of CP in sugarcane tops silage irrespective of sugarcane variety. Similarly, the CP content in whole crop maize silage, corn silage, sorghum silage, and the mixed silage of intercropping corn and alfalfa increased significantly with the increase in the N application rate (Islam et al., 2012; Safdarian et al., 2014; Farhadi et al., 2022; Xu et al., 2022). This is mainly related to the large amount of CP produced by the use of N in the environment before ensiling (Boddey et al., 2001). Additionally, similar to the present study, Ertekin et al. (2021) found that forage variety and nitrogen levels affect the OM content in Italian ryegrass silage. Moreover, in this study, at N application rates of both 0 and 150 kg/ha, the ash content of sugarcane tops silage produced from T11 was highest, while at N application rates of 300 kg/ha, the ash content of the sugarcane tops silage produced from C22 was lowest. Similarly, the ash content of annual ryegrass silage produced from the Baquend variety at N application rates of no more than 225 kg/ha and that of whole-crop maize silage produced from the KXA 7211 (harvested on October 6 and September 15) or Andante (harvested on October 27) varieties at N application rates of 33 kg/ha were the highest; however, the ash content of annual ryegrass silage produced from the KXA 7211 (harvested on October 6 and September 15) or Andante (harvested on October 27) varieties at N application rates of 300 kg/ha and that of whole-crop maize silage produced from the Tassilo variety at N application rates of 168 kg/ha were the lowest (Lynch et al., 2013; Assouma and Çelen, 2022). This is mainly attributed to the significant difference in the OM content in the forage before ensiling.

### 4.3 Fermentation quality of sugarcane tops silage

In the present study, for each variety, the pH of the group treated with 0 kg/ha N was greater than the pH of the groups treated with 150 kg/ha or 300 kg/ha N. The decreased silage pH was also found in millet silage after treatment with 112.5 kg/ha nitrogen and intercropped alfalfa–corn silage after treatment with 60 kg/ha nitrogen (Nematpour et al., 2020; Xu et al., 2022). This is mainly because the WSC content in fresh sugarcane tops after nitrogen application was higher than that in fresh sugarcane tops without nitrogen application, which provides more sugar substrates for lactic acid bacteria fermentation to produce lactic acid to reduce pH (Pahlow et al., 2003; Wang et al., 2019). However, possibly as a result of the differences in plant variety, the pH of cattle grass silage and millet–soybean silage increased with increasing rates of N application (Tremblay et al., 2005; Jahanzad et al., 2014). Moreover, in this study, at N application rates of 150 kg/ha and 300 kg/ha, the pH of sugarcane tops silage produced from B9 was significantly greater than that of sugarcane tops silage produced from C22. The reason for the higher silage pH could be that when the nitrogen application rate was 150 kg/ha or 300 kg/ha, the WSC content of fresh B9 sugarcane tops was lower than that of C22 before ensiling or that B9 sugarcane synthesizes more nitrate with its strong nitrogen fixation efficiency (Driehuis and Elferink, 2000; Yang et al., 2008; Zeng et al., 2020). Notably, the pH is an important parameter that reflects the degree of silage fermentation. Furthermore, in the present study, sugarcane tops silage treated with N contained more LA than that without N treatment. This is because the WSC content of fresh sugarcane tops after nitrogen application was higher than that before nitrogen application. This result was also reported by Li et al. (2016); Ertekin et al. (2021), and Xu et al. (2022). Meanwhile, in the present study, the LA content of sugarcane tops silage produced from C22 treated with N was significantly higher than that of sugarcane tops silage produced from B9 and T11. This is mainly because when the N application rate was 150 kg/ha, the WSC content of C22 sugarcane tops was highest compared to those two groups before ensiling. The lowest pH and highest LA content provide a comfortable environment for LAB to multiply (Pahlow et al., 2003). Therefore, in the current study, when the N application rate was 150 kg/ha, the LAB counts of sugarcane tops silage produced from C22 were also the highest. Similarly, Lynch et al. (2013) compared the effects of three whole-crop maize cultivars and two nitrogen application rates on their silage and found the lowest pH and highest LA content in whole-crop maize silage produced from the KXA 7211 variety (harvested on October 6) treated with 168 kg N/ha nitrogen. In general, high levels of lactic acid reflect effective fermentation and no dry matter loss from the silage. The AN content mainly reflects the degree of proteolysis (Lopez et al., 1970). In the present study, at a N application rate of 300 kg/ha, the AN content of sugarcane tops silage produced from T11 was lowest. Similar results were detected in Italian ryegrass silage, and the AN content of Italian ryegrass silage produced from the Devis and Hellen varieties was low (Ertekin et al., 2021). This result might be because at high N levels, the proteolysis in sugarcane tops silage produced from T11 is limited. However, in this study, at a N application rate of 300 kg/ha, the yeast counts of sugarcane tops silage produced from B9 were highest. Similar results were found in whole-crop maize silage produced from the Andante variety (harvested on October 6) treated with 33 kg N/ha nitrogen (Lynch et al., 2013). The result is possibly due to the highest pH of sugarcane tops silage being produced from the B9 variety at N application rates of both 150 and 300 kg/ha, making it difficult to effectively inhibit the growth and reproduction of yeast (Pahlow et al., 2003). Additionally, studies have shown that sugarcane variety B9 with strong nitrogen fixation ability can synthesize nitrate even in a low nitrogen environment, thereby inhibiting the growth and reproduction of *Clostridium* in silage (Pahlow et al., 2003; Yang et al., 2008; Zeng et al., 2020). Thus, in this study, at N application rates of both 0 and 150 kg/ha, the *Clostridium* counts of the sugarcane tops silage produced from B9 were the lowest. The presence of a large amount of yeast and *Clostridium* is an important factor for the secondary fermentation of silage, which in turn leads to the loss of silage nutrients.

### 4.4 Bacterial and fungal diversity of sugarcane tops silage after aerobic exposure

In this study, for all groups, the richness of bacterial community decreased in the early and middle stages of aerobic exposure, which was consistent with the findings of Zhang et al. (2019) and Wang et al. (2020). This might be due to the lack of nutrient substrates in silage (McGarvey et al., 2013). However, in this study, for all groups, the richness and diversity of fungal community decreased in the middle and later stages of aerobic exposure. A similar result was found by Wang et al. (2022), who reported that after 12 days of aerobic exposure, the richness and diversity of fungal community decreased in whole-crop wheat silage. Meanwhile, the richness of the fungal community also decreased in barley silage (Nair et al., 2019). The reason for this might be that the proliferation of acetic acid bacteria on aerobic exposure, which has an inhibitory effect on fungi (Driehuis and Van Wikselaar, 1996).

Additionally, in this study, in all treatment groups, the richness of the bacterial community increased in the middle and later stages of aerobic exposure. A similar result was found by Nair et al. (2019), who reported that after 21 days of aerobic exposure, the richness of the bacterial community increased in barley silage. A possible reason for this could be that with prolonged aerobic exposure, aerobic bacteria begin to proliferate in large numbers (Liu et al., 2019). However, in this study, the richness and diversity of the fungal community increased after 2 days of aerobic exposure. Wang et al. (2020) also reported that after 5 days of aerobic exposure, the richness and diversity of the fungal community increased in sugarcane tops silage treated or untreated with LAB inoculants. It may be that in the early stage of aerobic exposure, fungi could compete with lactic acid bacteria for the utilization of sugar substrates and decompose and use lactic acid in silage as their nutrient substrates to achieve proliferation (Driehuis and Elferink, 2000; Guo et al., 2018).

### 4.5 The bacterial community dynamics of sugarcane tops silage after aerobic exposure

In the current study, Proteobacteria and Firmicutes were the most abundant bacterial phyla on d 0–14 of aerobic exposure in all treatment groups. Consistent with this, Proteobacteria and Firmicutes were the most abundant bacterial phyla also found in natural sugarcane tops silage and maize silage after aerobic exposure, respectively (Xu et al., 2019; Zhang et al., 2019). Firmicutes are important acid-hydrolyzing microorganisms under anaerobic conditions, followed by Proteobacteria (Ogunade et al., 2018a). Moreover, in this study, the BL, BM, BH, and CL groups shifted from Proteobacteria to Firmicutes during early aerobic exposure, the BL and BH groups shifted from Proteobacteria to Firmicutes during the middle period of aerobic exposure, the CM and BH groups shifted from Proteobacteria to Firmicutes during the later period of aerobic exposure, and the CH group shifted from Proteobacteria to Firmicutes throughout the period of aerobic exposure. Similar results were also found in natural sugarcane tops silage, whole-crop wheat silage, and alfalfa silage after 3, 12, and 20 days aerobic exposure, respectively (McGarvey et al., 2013; Zhang et al., 2019; Wang et al., 2022). These results suggest that there are particular environmental conditions for the proliferation of Firmicutes, such as sufficient organic acids and lower pH (Hou et al., 2022). In contrast, during the middle period of aerobic exposure, the CM group shifted from Firmicutes to Proteobacteria and the BM, BL, and CL groups shifted from Firmicutes to Proteobacteria during the later period of aerobic exposure. These results corroborate the reports of Liu et al. (2020) and Ali et al. (2020). The explanation may be that with the extension of aerobic exposure, increases in pH or decreases in organic acids are not suitable for the proliferation of Firmicutes.

Furthermore, in this study, several Proteobacteria and Firmicutes genera underwent similar changes: *Lactobacillus*, *Escherichia-Shigella*, and *Clostridium* were the dominant bacterial genera on days 0–6 of aerobic exposure in all six groups. This was similar with previous studies, which identified *Lactobacillus* as the dominant bacterial taxon in triticale silage, corn silage, and barley silage after aerobic exposure (Duniere et al., 2017; Keshri et al., 2018; Liu et al., 2020). *Escherichia-Shigella* are facultative, anaerobic, Gram-negative bacteria belonging to the family Enterobacteriaceae that survive and multiply in poorly fermented silage (Dunière et al., 2013; Ogunade et al., 2016; Ogunade et al., 2017). Studies have shown that *Escherichia-Shigella* mainly produces Shiga toxin in silage and is an important factor in causing foodborne diseases (Driehuis et al., 2018). *Clostridium* is considered an undesirable bacterium in silage, especially protein-rich feed silage (Ni et al., 2017; Ni et al., 2018). This is because many strains of *Clostridium* thrive in humid environments under high pH (>5) and high ammonia content conditions (Oude Elferink et al., 2000). In silage, *Clostridium* causes protein degradation, increases dry matter loss, and produces butyric acid (Zheng et al., 2017). Over 14 d of aerobic exposure, the relative abundances of *Bacillus*, *Lysinibacilli*, and *Paenibacillus* in the sugarcane tops silage produced from variety C22 treated with high and medium levels of nitrogen, as well as in the sugarcane tops silage produced from variety B9 treated with high levels of nitrogen, increased gradually, with these genera eventually dominating the bacterial community. This was consistent with the results of Peng et al. (2018), who reported that, after 14 d aerobic exposure, *Bacillus*, *Lysinibacilli*, and *Paenibacillus* were the dominant bacterial taxa in purple prairie clover silage with or without PEG treatment. Similarly, Duniere et al. (2017) showed that in oat, triticale, barley, and intercropped silages, the relative abundances of *Bacillus* increased significantly and became dominant after 14 d of aerobic exposure. A previous study showed that some *Bacillus* species can improve the aerobic stability of silage by producing bacteriocin (Zhang et al., 2022). In contrast, in the BH, CM, and CH groups, the relative abundance of *Acinetobacter* increased sharply after 14 d of aerobic exposure. Consistent with this, Liu et al. (2019) reported that, as aerobic exposure was prolonged, the relative abundance of *Acinetobacter* in barley silage increased. *Acinetobacter* is an aerobic, non-fermenting bacterium that can survive in an anaerobic environment with acetate as a substrate but requires energy from the degradation of soluble carbohydrates (Muraro et al., 2021).

### 4.6 The fungal community dynamics of sugarcane tops silage after aerobic exposure

In the present study, Ascomycota and Basidiomycota were the most abundant fungal phyla at any time point in all groups after aerobic exposure. Similar results have been reported in silage from sugarcane tops (Zhang et al., 2019), purple prairie clover (Peng et al., 2018), and barley (Liu et al., 2019). Moreover, after aerobic exposure, the relative abundance of Ascomycota increased and that of Basidiomycota decreased in all six treatment groups. Similar results were also detected in barley silage, sugarcane top silage, and whole-crop wheat silage (Liu et al., 2019; Zhang et al., 2019; Wang et al., 2022). Ascomycetes and Basidiomycetes are the main groups involved in cellulolysis, and both are diverse and species-rich phyla in the fungal kingdom.

Additionally, in this study, *Pleurotus* and *Fusarium* were the dominant fungal genus in all groups before aerobic exposure. Consistent with this, García-Fontana et al. (2016) reported that *Fusarium* was the main fungal taxon in wheat and maize silage. *Fusarium* can secrete a variety of mycotoxins but does not produce mycotoxins when grown rapidly at temperatures between 25°C and 30°C (Ogunade et al., 2018b). Motomura et al. (2003) reported that *Pleurotus* sp. can secrete enzymes that degrade aflatoxin B1, and it works best in an environment of pH 4.0–5.0. Moreover, in this study, *Candida* was the dominant fungal genus in all six groups after 2, 4, 6, and 14 d of aerobic exposure, while *Pichia* was the dominant fungal genus in all six groups after 6 and 14 d of aerobic exposure. Similarly, *Candida* and *Pichia* were the main fungi in alfalfa silage during aerobic exposure (Bai et al., 2020; Jiang et al., 2020). Yeasts that assimilate lactic acid, such as *Candida* and *Pichia*, are usually the primary flora performing the aerobic degradation of silage, because these taxa resist acids and increase silage pH by consuming organic acids (Driehuis and Elferink, 2000). In addition, in the current study, after 14 days of aerobic exposure, the relative abundance of *Monascus* suddenly increased in the BM, BH, and CM groups. Similarly, Jiang et al. (2020) reported that the relative abundance of *Monascus* increased in alfalfa silage after 14 days of air exposure, with or without the addition of *Pediococcus pentosaceus*. Moreover, in this study, the greatest relative abundance of *Monascus* found in the BH group and over than 50% at 14 d of aerobic exposure. Citrinin, a secondary metabolite of *Monascus*, causes functional and structural kidney damage as well as liver metabolism changes (Schneweis et al., 2001).

### 4.7 The correlations between bacterial and fungal community in sugarcane tops silage after aerobic exposure

In this study, *Monascus* was positively correlated with *Acinetobacter*, *Lysinibacillus*, *Bacillus*, and *Paenibacillus*, irrespective of N level and sugarcane varieties. Previous studies have shown that ester-producing fungi and lactic acid-producing bacteria have a synergistic effect, converting lactic acid and alcohol into ethyl lactate (Li et al., 2019). *Monascus* is considered a common microorganism that develops in the late stages of the aerobic degradation of silage; this fungus also has strong esterification abilities in the late fermentation stage (Driehuis and Elferink, 2000; Zheng et al., 2011). The genera *Bacillus*, *Paenibacillus*, and *Lysinibacillus* belong to the same order Bacillales. Previous studies indicated that many species from these genera not only have anti-fungal properties but also have the ability to produce lactic acid (Ferchichi et al., 2019; Ribeiro et al., 2021). Thus, the lactic acid produced by these bacteria may provide abundant raw materials for the esterification reaction of *Monascus*. Additionally, Henein et al. (2016) reported that *Acinetobacter* sp. produced L-lactic, which is one of the key sources of carbon and energy for *Acinetobacter* sp. growth. However, *Acinetobacter* is considered harmful and may cause the aerobic degradation of silage (Liu et al., 2013). Moreover, in this study, *Halomonas* was positively correlated with *Monascus* in silage produced from variety B9, irrespective of nitrogen level. The reason for this could be that, owing to its unique halophilic characteristics, *Halomonas* can not only grow in a high pH environment, but it can also decompose and utilize nitrate by secreting nitrate reductase (Wang et al., 2017). However, in the present study, *Halomonas* was negatively correlated with *Monascus* in silage produced from variety C22, irrespective of nitrogen level. This can be explained by the competitive utilization of substrate organic acids, as *Halomonas* is similar to *Monascus* in that it has a strong ability to consume lactic acids to produce esters after aerobic exposure (Yue et al., 2014; Chen et al., 2018). Additionally, in the present study, *Dekkera* was positively correlated with *Acinetobacter*, *Lysinibacillus*, *Paenibacillus*, and *Gluconacetobacter* at low levels of nitrogen in silage produced from varieties B9 and C22. Previous studies have shown that at pH 4.5, *Dekkera* fails to utilize all the glucose initially present in the medium while still producing a small amount of acetic acid (Freer et al., 2003). Therefore, it is possible that the non-competitive use of this sugar substrate, coupled with the production of acetic acid, creates an acidic environment that allows *Dekkera* to achieve a symbiosis with those two bacteria. In contrast, in this study, *Dekkera* was negatively correlated with *Acinetobacter* and *Gluconacetobacter* at high and medium levels of nitrogen in silage produced from varieties B9 and C22. The reason for this may be that *Dekkera* yeast competes with *Acinetobacter* and *Gluconacetobacter* for the utilization of sugar substrates (such as glucose and maltose) to produce acetate under aerobic conditions (Blomqvist et al., 2010).

## 5 Conclusions

This study analyzed the effects of nitrogen application level and sugarcane variety on the fermentation quality and microbial community of sugarcane tops silage. The results showed that after nitrogen fertilization, sugarcane variety B9, with strong nitrogen fixation ability, can obtain more CP in sugarcane tops silage with increased nitrogen fertilization, inhibit *Clostridium* proliferation, and is more likely to breed yeast; however, it has no positive effect on other nutrients or fermentation parameters in silage. On the contrary, after reasonable nitrogen fertilization, sugarcane variety C22, with poor nitrogen fixation ability, can obtain sugarcane tops silage with higher DM content, more LAB counts, reasonable chemical composition, and better fermentation quality. However, these results were not found in sugarcane tops silage produced from sugarcane variety T11, with no nitrogen fixation ability, whether it was treated with nitrogen or not, despite the inhibition of proteolysis in silage at high doses of nitrogen. Additionally, under air conditions, sugarcane tops silage produced from the nitrogen treatment of sugarcane variety B9, with strong nitrogen fixation ability, and from sugarcane variety C22, with poor nitrogen fixation ability, after treatment at high nitrogen rates are more conducive to enhancing *Monascus* abundance, while sugarcane tops silage produced from the nitrogen treatment of sugarcane variety C22, with poor nitrogen fixation ability, and from sugarcane variety B9, with strong nitrogen fixation ability, after treatment at high nitrogen rates are more conducive to enhancing *Bacillus* abundance. However, the correlation analysis showed that *Monascus* was positively correlated with *Bacillus*, irrespective of nitrogen level and sugarcane variety. Therefore, considered comprehensively, sugarcane variety C22 with a nitrogen application rate of 150 kg/ha is the best choice for obtaining high-quality sugarcane tops silage.

## Data availability statement

Bacterial and fungal raw sequencing data have been deposited in the NCBI (https://www.ncbi.nlm.nih.gov/) BioProject database, with accession numbers PRJNA895585 and PRJNA895721, respectively.

## Author contributions

QG, LZ, and CZ carried out the experimental design. QG, LZ, XZ, and CZ performed the experiments. QG, LZ, XZ, BL, and CZ analyzed the experimental data. QG wrote the first draft. QG and CZ revised the manuscript. All authors contributed to the article and approved the submitted version.
